# Associations Between Circulating Insulin-Like Growth Factor 1 and Mortality in Women With Invasive Breast Cancer

**DOI:** 10.3389/fonc.2020.01384

**Published:** 2020-08-19

**Authors:** Yifei Zhu, Tiange Wang, Jiayi Wu, Ou Huang, Li Zhu, Jianrong He, Yafen Li, Weiguo Chen, Xiaosong Chen, Kunwei Shen

**Affiliations:** ^1^Comprehensive Breast Health Center, Ruijin Hospital, Shanghai Jiao Tong University School of Medicine, Shanghai, China; ^2^Shanghai Institute of Endocrine and Metabolic Diseases, Ruijin Hospital, Shanghai Jiao Tong University School of Medicine, Shanghai, China

**Keywords:** breast cancer, insulin-like growth factor 1, mortality, breast cancer-specific mortality, clinical risk factor

## Abstract

**Background:** Studies on the association between circulating insulin-like growth factor 1 (IGF1) and prognosis of breast cancer are limited. Whether this association is modified by insulin levels and clinical characteristics is unclear.

**Methods:** Serum concentrations of IGF1 as well as IGF binding protein 3 (IGFBP3), IGF1/IGFBP3 ratio, insulin, and C-peptide were prospectively examined in 2,682 invasive breast cancer patients who received surgery in Ruijin Hospital, Shanghai, between 2012 and 2017. Cox proportional hazards models were used to calculate hazard ratios (HRs) and 95% confidence intervals (CIs) for all-cause mortality, breast cancer-specific mortality, and breast cancer recurrence associated with different levels of IGF1 and other biomarkers with multivariable adjustment.

**Results:** Compared with patients with low IGF1, patients with high IGF1 had a significantly lower risk of all-cause mortality (HR, 0.53; 95% CI, 0.29–0.96) and a borderline lower risk of breast cancer-specific mortality (HR, 0.53; 95% CI, 0.27–1.02). The inverse association between IGF1 and all-cause mortality was consistent across stratification subgroups but was more pronounced among patients with high insulin (HR, 0.40; 95% CI, 0.18–0.89), were premenopausal (HR, 0.34; 95% CI, 0.12–0.97), with a tumor size >2 cm (HR, 0.35; 95% CI, 0.17–0.73), with positive lymph node (HR, 0.49; 95% CI, 0.25–0.98), and with a high Ki-67 level (HR, 0.49; 95% CI, 0.26–0.95) (all P for interaction >0.05). No significant associations were found for IGFBP3, IGF1/IGFBP3 ratio, insulin, and C-peptide levels with all-cause mortality, breast cancer-specific mortality, and breast cancer recurrence.

**Conclusion:** Circulating IGF1 was inversely and independently associated with all-cause mortality in invasive breast cancer patients, and this association was consistent across clinical risk factors.

## Introduction

The insulin-like growth factors (IGFs) are multifunctional peptides that regulate proliferation, survival, differentiation, and apoptosis for a diverse range of normal and malignant cells (1, 2). IGF1 is highly homologous with insulin, and the biological activity of IGF1 is regulated by IGF binding proteins (IGFBPs) (3, 4). IGF1 plays a critical role in both physiology and pathological conditions (3–7). Physiologically, IGF1 has beneficial properties in maintaining genetic stability, controlling lipid and glucose metabolism, and protecting liver, and cardiovascular function (5). Pathologically, IGF1 is a major regulator in the development, progression, and metastasis of several malignant cancers (6, 7). The concentrations of biomarkers of the IGF axis can be measured easily in blood and might therefore be useful in the prediction of risk for specific cancers.

Breast cancer is an endocrine-related cancer. Previous studies have indicated that high concentrations of IGF1 were associated with an increased risk of breast cancer, especially for premenopausal breast cancer and estrogen receptor-positive breast cancer (8, 9). However, studies on the associations between circulating IGF1 and prognosis of breast cancer are scarce and have shown inconsistent results (10–13). In a multiethnic, prospective cohort study of 600 women diagnosed with stage I–IIIA breast cancer, high serum levels of IGF1, and IGF1/IGFBP3 ratio were associated with increased risk of all-cause mortality (10), whereas, the majority of other studies did not support an association of higher levels of IGF1 and IGFBP3 with adverse prognosis of breast cancer such as all-cause mortality, breast cancer-specific mortality, and breast cancer recurrence (11–13). These inconsistent findings may be partly due to the heterogeneity of study populations, the bias introduced by therapy strategies of breast cancer, and the influence of potential modifiers or confounders. Specifically, the concentrations of IGF1 varied significantly between individuals, which may also affect the consistence of study findings in different subgroups. In addition, whether the cross talk between insulin and IGF signaling pathways may influence the associations between biomarkers of the IGF axis and the prognosis of breast cancer is unclear.

In this study, taking advantage of a large prospective cohort study, we examined the associations of IGF1 as well as other biomarkers of insulin and the IGF axis including insulin, C-peptide, IGFBP3, and the IGF1/IGFBP3 ratio with all-cause mortality, breast cancer-specific mortality, and breast cancer recurrence in women with invasive breast cancer.

## Methods

### Study Setting and Patients

Women who were diagnosed with invasive breast cancer were enrolled from the Comprehensive Breast Health Center, Ruijin Hospital, Shanghai Jiao Tong University School of Medicine, between 2012 and 2017. Stored hematoxylin and eosin-stained sections from all patients were evaluated by experienced pathologists in the Department of Pathology, Ruijin Hospital. Breast cancer was diagnosed by histopathologic examination according to the fourth edition of the World Health Organization Classification of Tumors of the Breast (14). Patients who met the inclusion criteria were included in the analysis: (1) invasive breast cancer; (2) T1–T3; (3) lymph node involvement N0–N3; (4) non-metastasis (M0); and (5) with complete data available on clinical information and IGF1 levels. This analysis included 2,682 patients. All study patients provided written informed consent. The study protocol was approved by the Medical Ethics Committee of Ruijin Hospital, Shanghai Jiao Tong University.

### Clinical Data Collection

Standardized clinical data of patients with breast cancer were obtained from Shanghai Jiao Tong University Breast Cancer Database. Clinical information includes demographic characteristics, body mass index (BMI), menopausal status, family history of breast cancer, molecular subtype, TNM stage, tumor size, histology of breast cancer, lymph node status, estrogen receptor (ER) status, progesterone receptor (PR) status, human epidermal growth factor receptor 2 (HER2) status, Ki-67 level, and adjuvant therapies. Weight was measured to the nearest 0.1 kg wearing light indoor clothing, and height was measured to the nearest 0.1 cm without shoes. BMI was calculated as weight in kilograms divided by height in meters squared.

Immunohistochemical (IHC) assessment of ER, PR, HER2, and Ki-67 was performed in the Department of Pathology, Ruijin Hospital, Shanghai Jiaotong University School of Medicine. Pathological diagnosis of breast cancer was confirmed by an oncologist, with a random sample of records reviewed by a second oncologist. Patients were classified as having stage I–III breast cancer based on American Joint Committee on Cancer (AJCC) stage of disease classification (Eighth Edition) (15). ER and PR status of tumors was categorized as positive (≥1% positive invasive cell nuclear staining) or negative (<1% positive invasive cell nuclear staining). According to the 2018 American Society of Clinical Oncology (ASCO)/College of American Pathologists (CAP) guidelines, HER2-positive status was defined as IHC HER2 3+ or fluorescence *in situ* hybridization (FISH) HER2 amplified. For Ki-67 expression scoring, 14% was selected as the cutoff value in determining low or high status. According to the definitions of intrinsic subtypes of the 2013 St. Gallen breast cancer consensus (16), five breast cancer molecular subtypes were classified: Luminal A (ER+/HER2-, Ki67 <14% and PR ≥20%), Luminal B-HER2- (ER+/HER2-, Ki67 ≥14 % or ER+/HER2-, PR <20%, or ER-/PR+/HER2-), Luminal B-HER2+ (ER+/any PR/HER2+), TN (ER-/PR-/HER2-), and HER2+ (ER-/PR-/HER2+). Adjuvant therapies included chemotherapy, radiotherapy, endocrine therapy, and targeted therapy.

### Biomarker Measurement

After an overnight fast of at least 8 h, blood samples were collected from each patient before the surgical procedure. Plasma IGF1 and IGFBP3 were measured by chemiluminescent immunoassay using IMMULITE 2,000 system (Siemens AG, Munich, Germany). The IGF1/IGFBP3 ratio was calculated. Serum insulin and C-peptide were measured by electrochemiluminescence immunoassay on Cobas E601 analyzers (Hoffman-La Roche Ltd., Basel, Switzerland). All biomarkers were examined at the Department of Clinical Laboratory, Ruijin Hospital.

### Survival Outcomes

The prognosis of breast cancer was collected by telephone or clinic visits every 12 months during the follow-up period. All-cause mortality was defined as death from any cause during the follow-up. Breast cancer-specific mortality was defined as death from breast cancer, with the same intervals as for all-cause mortality. Recurrence was defined as invasive ipsilateral breast tumor recurrence, local/regional invasive recurrence, distant recurrence, ipsilateral ductal carcinoma *in situ* (DCIS), and contralateral DCIS (17).

### Statistical Analysis

Concentrations of IGF1, IGFBP3, IGF1/IGFBP3 ratio, insulin, and C-peptide were categorized into low and high levels using median cutoff points (Supplementary Table 1). Baseline characteristics of the overall study patients and patients by IGF1 levels were presented as mean (standard deviation) for continuous variables or number (proportion) for categorical variables. The baseline characteristics of patients within low and high levels of IGF1 were compared by analysis of variance for continuous variables and by chi-square test for categorical variables.

In the time-to-event analysis for mortality, patients were censored at the date of death or the end of follow-up, whichever occurred first. In the time-to-event analysis for breast cancer recurrence, patients were censored at the date of breast cancer recurrence or the end of follow-up, whichever occurred first. Person-time was calculated from the enrollment date to the censoring date for each patient. Cox proportional hazards models were used to calculate hazard ratios (HRs) and 95% confidence intervals (CIs) for all-cause mortality, breast cancer-specific mortality, and breast cancer recurrence associated with high levels of biomarkers of insulin and the IGF axis in comparison with respective low levels. Models were adjusted for age, BMI, menopausal status, tumor size, lymph node status, chemotherapy, radiotherapy, endocrine therapy, and targeted therapy. Kaplan–Meier survival curves were used to estimate the cumulative incidence of all-cause mortality, breast cancer-specific mortality, and breast cancer recurrence according to IGF1 levels. For exploring analyses, the HRs and Kaplan–Meier survival curves of post-recurrence mortality according to IGF1 levels were also examined.

Stratification analyses were performed to evaluate the association between IGF1 and all-cause mortality across subgroups of age (<50 or ≥50 years), BMI (<25 or ≥25 kg/m^2^), menopausal status (premenopausal or postmenopausal status), tumor size (≤2 cm or >2 cm), lymph node (positive or negative), ER status (positive or negative), PR status (positive or negative), HER2 overexpressing (yes or no), luminal (yes or no), triple-negative (yes or no), and Ki-67 level (low or high). Interactions between IGF1 and insulin on all-cause mortality, breast cancer-specific mortality, and breast cancer recurrence were further analyzed to examine the effect modification of insulin on the association between IGF1 and prognosis of breast cancer. Multiplicative interactions were tested by including the product term (IGF1 level × insulin level) as well as the main association in the models.

All statistical analyses were performed by using SAS software, version 9.2 (SAS Institute). All reported *P*-values are nominal and two-sided, and a *P* < 0.05 was considered statistically significant.

## Results

### Baseline Characteristics

Baseline characteristics of study patients by low and high IGF1 levels are shown in Table 1. Of 2,682 women included in this study, the mean age (SD) was 55.3 (12.4) years. Compared with patients with low IGF1, patients with high IGF1 were younger (50.7 vs. 59.9 years), had lower levels of BMI (23.1 vs. 23.7 kg/m^2^), had lower proportions of being postmenopausal (46.3 vs. 74.6%), and had relatively higher levels of IGFBP3 (4.4 vs. 3.6 μg/ml), IGF1/IGFBP3 ratio (48.8 × 10^−3^ vs. 33.0 × 10^−3^), insulin (9.4 vs. 9.0 μIU/ml), and C-peptide (2.2 vs. 2.1 μg/L; all *P* ≤ 0.04). Patients with high IGF1 were more likely to have a high Ki-67 level (67.3 vs. 62.2%) and receive chemotherapy (76.4 vs. 65.2%), radiotherapy (57.4 vs. 51.7%), and targeted therapy (21.7 vs. 18.1%; all *P* ≤ 0.02).

**Table 1:** Baseline characteristics of study patients^a^.

**Baseline characteristic**	**Overall**	**IGF1**		
		**Low**	**High**	***P*-value**
Number of patients	2,682	1,329	1,353	–
Age, years	55.3 (12.4)	59.9 (11.6)	50.7 (11.3)	<0.0001
Body mass index, kg/m^2^	23.4 (3.2)	23.7 (3.5)	23.1 (3.0)	<0.0001
Postmenopausal status, *n* (%)	1,617 (60.3)	991 (74.6)	626 (46.3)	<0.0001
**Insulin and IGF axis biomarker**				
IGF1, ng/ml	164.7 (62.4)	115.9 (27.7)	212.6 (48.3)	<0.0001
IGFBP3, μg/ml	4.0 (0.9)	3.6 (0.8)	4.4 (0.9)	<0.0001
IGF1/IGFBP3 ratio, × 10^−3^	40.9 (12.2)	33.0 (7.6)	48.8 (10.7)	<0.0001
Insulin, μIU/ml	9.2 (5.1)	9.0 (5.5)	9.4 (4.7)	0.03
C-peptide, μg/L	2.1 (0.8)	2.1 (0.8)	2.2 (0.7)	0.04
**Clinical characteristic**				
**TNM stage**, ***n*** **(%)**				
I	1,147 (44.0)	575 (44.1)	572 (43.8)	0.05
II	1,136 (43.5)	546 (41.9)	590 (45.2)	
III	326 (12.5)	182 (14.0)	144 (11.0)	
**Tumor size**, ***n*** **(%)**				
≤ 2 cm	1,498 (57.4)	734 (56.3)	764 (58.5)	0.26
>2 cm	1,111 (42.6)	569 (43.7)	542 (41.5)	
**Lymph node**, ***n*** **(%)**				
Positive	909 (33.9)	450 (33.9)	459 (33.9)	0.97
Negative	1,773 (66.1)	879 (66.1)	894 (66.1)	
**ER**, ***n*** **(%)**				
Positive	1,962 (73.2)	986 (74.2)	976 (72.1)	0.23
Negative	720 (26.9)	343 (25.8)	377 (27.9)	
**PR**, ***n*** **(%)**				
Positive	1,607 (59.9)	795 (59.8)	812 (60.0)	0.92
Negative	1,075 (40.1)	534 (40.2)	541 (40.0)	
**Molecular subtype**, ***n*** **(%)**				
HER2 overexpressing	334 (12.5)	167 (12.6)	167 (12.3)	0.20
Luminal A	550 (20.5)	275 (20.7)	275 (20.3)	
Luminal B (HER2 positive)	289 (10.8)	129 (9.7)	160 (11.8)	
Luminal B (HER2 negative)	1,110 (41.4)	575 (43.3)	535 (39.5)	
Triple-negative breast cancer	373 (13.9)	171 (12.9)	202 (14.9)	
Other	26 (1.0)	12 (0.9)	14 (1.0)	
**Ki-67 level**, ***n*** **(%)**				
Low (<14)	944 (35.2)	502 (37.8)	442 (32.7)	0.006
High (≥14)	1,736 (64.8)	826 (62.2)	910 (67.3)	
**Adjuvant therapy**				
Chemotherapy, *n* (%)	1,900 (70.8)	866 (65.2)	1,034 (76.4)	<0.0001
Radiotherapy, *n* (%)	1,463 (54.6)	687 (51.7)	776 (57.4)	0.003
Endocrine therapy, *n* (%)	1,908 (71.1)	959 (72.2)	949 (70.1)	0.25
Targeted therapy, *n* (%)	535 (20.0)	241 (18.1)	294 (21.7)	0.02

a *Data are mean (standard deviation) for continuous variables, or number (%) for categorical variables. The number of missing values was 849 for IGFBP3, 849 for IGF1/IGFBP3 ratio, 73 for TNM stage, and 2 for Ki-67. Percentages may not add up to 100% due to rounding*.

*ER, estrogen receptor; HER2, human epidermal growth factor receptor 2; IGF1, insulin-like growth factor 1; IGFBP3, insulin-like growth factor binding protein 3; PR, progesterone receptor*.

### Association Between Biomarkers of Insulin and Insulin-Like Growth Factor Axis and Mortality

During a mean follow-up of 3.1 years (median = 3.0 years; range = 0–6.2 years; 8,284 person-years), 55 all-cause mortality, 43 breast cancer-specific mortality, and 157 breast cancer recurrence were documented (Supplementary Table 2). After multivariable adjustment for baseline demographic and clinical characteristics, patients with high IGF1 had a significantly lower risk of all-cause mortality (HR, 0.53; 95% CI, 0.29–0.96) and a borderline lower risk of breast cancer-specific mortality (HR, 0.53; 95% CI, 0.27–1.02) compared with patients with low IGF1 (Table 2). No other significant associations with all-cause mortality, breast cancer-specific mortality, and breast cancer recurrence were observed for IGFBP3, IGF1/IGFBP3 ratio, insulin, and C-peptide.

**Table 2:** Associations of biomarkers of insulin and IGF axis with all-cause mortality, breast cancer-specific mortality, and breast cancer recurrence^a^.

**Category**	**All-cause mortality**			**Breast cancer-specific mortality**			**Breast cancer recurrence**		
	**Person-years**	**Cases**	**HR (95% CI)^b^**	**Person-years**	**Cases**	**HR (95% CI)^b^**	**Person-years**	**Cases**	**HR (95% CI)^b^**
**IGF1**									
Low	4,001	37	1.00	4,001	28	1.00	3,891	77	1.00
High	4,283	18	0.53 (0.29–0.96)	4,283	15	0.53 (0.27–1.02)	4,138	80	1.01 (0.72–1.41)
**IGFBP3**									
Low	2,173	11	1.00	2,173	8	1.00	2,109	46	1.00
High	2,307	10	1.15 (0.48–2.77)	2,307	6	0.88 (0.30–2.60)	2,246	38	0.82 (0.53–1.27)
**IGF1/IGFBP3 ratio**									
Low	2,239	15	1.00	2,239	11	1.00	2,180	43	1.00
High	2,240	6	0.53 (0.20–1.41)	2,240	3	0.33 (0.09–1.23)	2,174	41	1.02 (0.65–1.60)
**Insulin**									
Low	4,257	23	1.00	4,257	22	1.00	4,108	89	1.00
High	4,027	32	1.21 (0.67–2.18)	4,027	21	0.90 (0.47–1.74)	3,920	68	0.74 (0.52–1.06)
**C-peptide**									
Low	4,183	23	1.00	4,183	22	1.00	4,055	77	1.00
High	4,101	32	1.14 (0.63–2.08)	4,101	21	0.87 (0.45–1.70)	3,973	80	1.06 (0.75–1.51)

a *There were 2,682 patients included in the analysis. The number of missing values was 849 for IGFBP3 and 849 for IGF1/IGFBP3 ratio*.

b *Data were adjusted for age, BMI, menopausal status (yes or no), tumor size (≤2 cm or >2 cm), lymph node status (positive or negative), chemotherapy (yes or no), radiotherapy (yes or no), endocrine therapy (yes or no), and targeted therapy (yes or no)*.

*BMI, body mass index; HR, hazard ratio; IGF1, insulin-like growth factor 1; IGFBP3, insulin-like growth factor binding protein 3*.

**Figure 1:**
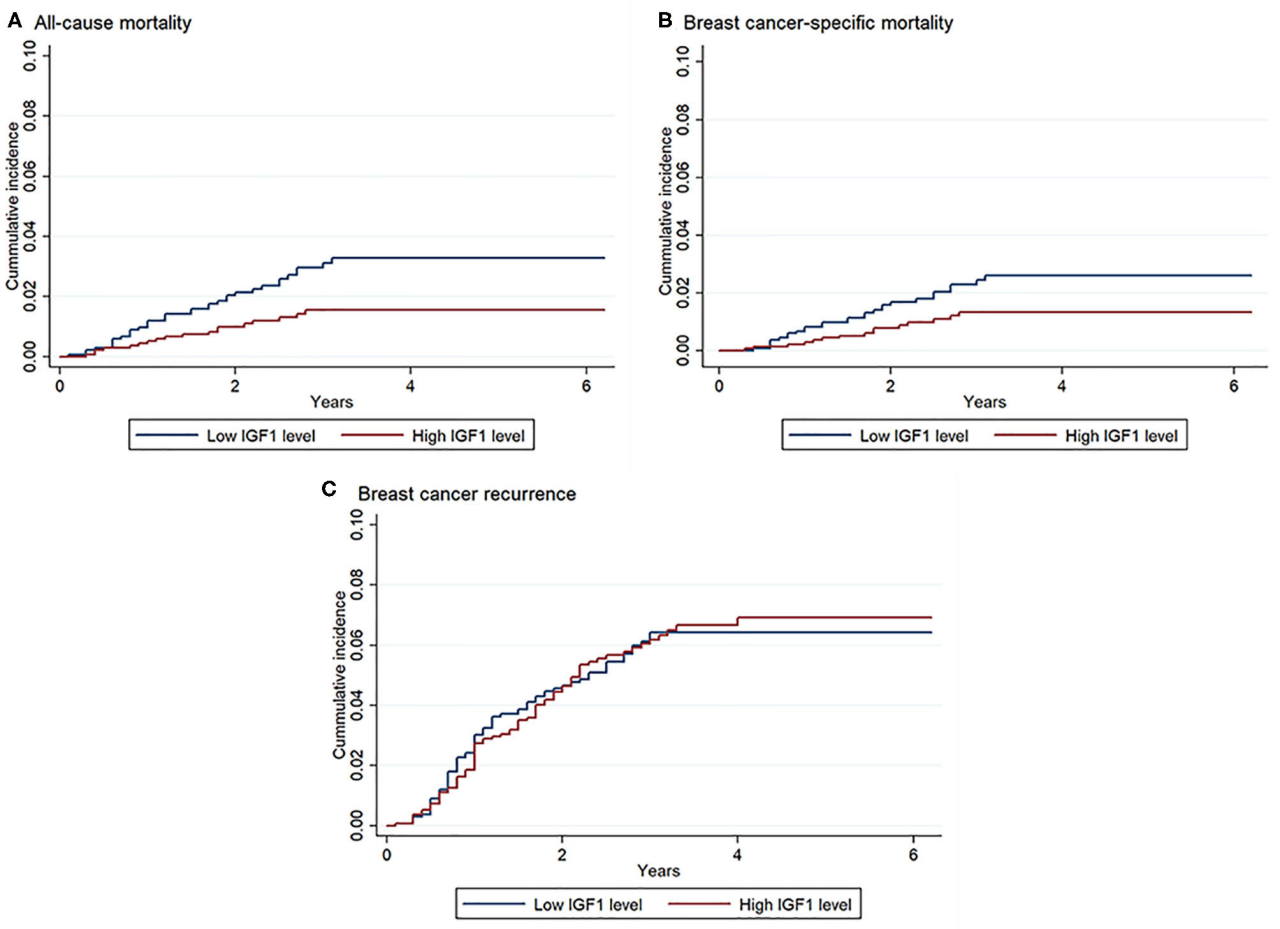
Kaplan–Meier curves of all-cause mortality, breast cancer-specific mortality, and breast cancer recurrence according to insulin-like growth factor 1 (IGF1) levels. There were 2,682 patients included in the analysis. Log-rank test: *P* = 0.007 for all-cause mortality, *P* = 0.038 for breast cancer-specific mortality, *P* = 0.90 for breast cancer recurrence. **(A)** Association between IGF1 and all-cause mortality; **(B)** Association between IGF1 and breast cancer-specific mortality; **(C)** Association between IGF1 and breast cancer recurrence.

As shown in Figure 1, unadjusted Kaplan–Meier curves identified that compared with patients with low IGF1, patients with high IGF1 exhibited lower cumulative incidence of all-cause mortality (1.3 vs. 2.8%, *P* = 0.007) and breast cancer-specific mortality (1.1 vs. 2.1%, *P* = 0.038). No statistically significant difference between high and low IGF1 on breast cancer recurrence was observed.

### Association Between Insulin-Like Growth Factor 1 and Mortality Across Stratification Subgroups

The association between IGF1 and all-cause mortality was consistent across insulin levels (Table 3). The interaction between IGF1 and insulin on all-cause mortality was not statistically significant (*P* for interaction = 0.20). The inverse association between IGF1 and all-cause mortality was more pronounced among patients with high insulin (HR, 0.40; 95% CI, 0.18–0.89), but was not significant among patients with low insulin (HR, 0.76; 95% CI, 0.31–1.87).

**Table 3:** Interactions between IGF1 and insulin on all-cause mortality, breast cancer-specific mortality, and breast cancer recurrence^a^.

**Category**	**All-cause**				**Breast cancer-specific**				**Breast cancer**			
	**mortality**				**mortality**				**recurrence**			
	**Person-**	**Cases**	**HR (95% CI)^b^**	***P* for**	**Person-**	**Cases**	**HR (95% CI)^b^**	***P* for**	**Person-**	**Cases**	**HR (95% CI)^b^**	***P* for**
	**years**			**interaction**	**years**			**interaction**	**years**			**interaction**
**Low insulin**												
Low IGF1	2,209	15	1.00		2,209	14	1.00		2,142	45	1.00	
High IGF1	2,048	8	0.76 (0.31–1.87)	0.20	2,048	8	0.77 (0.31–1.92)	0.27	1,966	44	1.36 (0.86–2.15)	0.29
**High insulin**												
Low IGF1	1,792	22	1.00		1,792	14	1.00		1,748	32	1.00	
High IGF1	2,234	10	0.40 (0.18–0.89)		2,234	7	0.41 (0.15–1.09)		2,172	36	0.85 (0.50–1.45)	

a *There were 2,682 patients included in the analysis*.

b *Data were adjusted for age, BMI, menopausal status (yes or no), tumor size (≤2 cm or >2 cm), lymph node status (positive or negative), chemotherapy (yes or no), radiotherapy (yes or no), endocrine therapy (yes or no), and targeted therapy (yes or no)*.

*BMI, body mass index; HR, hazard ratio; IGF1, insulin-like growth factor 1*.

**Figure 2:**
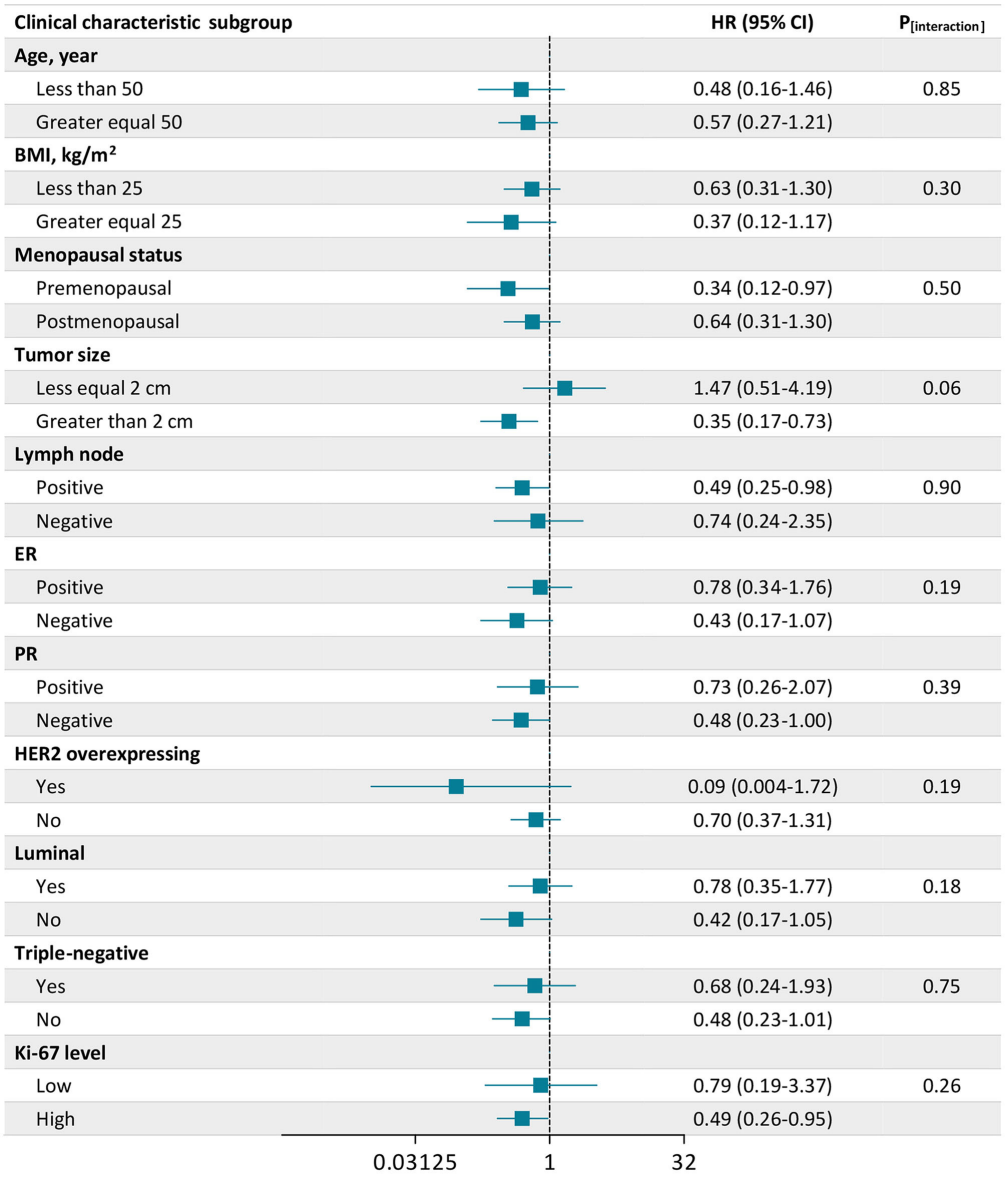
Association of insulin-like growth factor 1 (IGF1) with all-cause mortality according to subgroups of clinical characteristics. There were 2,682 patients included in the analysis. The number of missing values was 2 for Ki-67. Plots (bars) are hazard ratios (HRs) (95% CIs) for all-cause mortality associated with a high level of IGF1 compared with a low level of IGF1. Data were adjusted for age, body mass index (BMI), menopausal status (yes or no), tumor size (≤2 cm or >2 cm), lymph node status (positive or negative), chemotherapy (yes or no), radiotherapy (yes or no), endocrine therapy (yes or no), and targeted therapy (yes or no).

Figure 2 shows the association between IGF1 and all-cause mortality stratified by clinical characteristics. The association between high IGF1 and the decreased risk of all-cause mortality was consistent across stratifications by age, BMI, menopausal status, lymph node, ER status, PR status, HER2 overexpressing status, luminal status, triple-negative status, and Ki-67 level (all P for interaction ≥0.18). But the inverse association between IGF1 and all-cause mortality seemed to be more pronounced among patients who were premenopausal (HR, 0.34; 95% CI, 0.12–0.97), with a tumor size >2 cm (HR, 0.35; 95% CI, 0.17–0.73), with positive lymph node (HR, 0.49; 95% CI, 0.25–0.98), and with a high Ki-67 level (HR, 0.49; 95% CI, 0.26–0.95). Divergent associations of IGF1 with all-cause mortality were observed between tumor size ≤2 cm and >2 cm, with a borderline significant interaction (*P* for interaction = 0.06).

### Association Between Biomarkers of Insulin and Insulin-Like Growth Factor Axis and Post-recurrence Mortality

There were 38 mortalities (all were breast cancer-specific mortality) after the recurrence of breast cancer (*n* = 157). In exploring analyses, both high IGF1 (HR, 0.41; 95% CI, 0.20–0.84) and high IGF1/IGFBP3 ratio (HR, 0.21; 95% CI, 0.05–0.84) were associated with a lower risk of post-recurrence mortality (Supplementary Table 3). Consistently, patients with high IGF1 (15.0 vs. 33.8%, *P* = 0.006) or high IGF1/IGFBP3 ratio (7.3 vs. 25.6%, *P* = 0.021) had lower cumulative incidence of post-recurrence mortality (Supplementary Figure 1).

## Discussion

In this study, higher concentrations of circulating IGF1 were significantly and independently associated with a lower risk of all-cause mortality in women with invasive breast cancer. The inverse association between IGF1 and all-cause mortality remained consistent across stratification subgroups of clinical risk factors, and such association was more prominent among patients who were premenopausal, with high insulin, with a tumor size >2 cm, with positive lymph node, and with a high Ki-67 level.

Emerging evidence supports the key growth regulatory role of the IGF system in the development of breast cancer (18–20). A growing body of epidemiological investigations has examined the association between IGF1 and the incidence of breast cancer (8, 9). A meta-analysis of 21 case-control studies including 3,609 cases and 7,137 controls has found that high concentrations of IGF1 and IGFBP3 were associated with an increased risk of incident breast cancer among premenopausal women but not among postmenopausal women (8). A pooled data analysis of 17 prospective studies has further confirmed the positive association between circulating IGF1 and breast cancer risk, which was not substantially modified by IGFBP3 and did not differ markedly by menopausal status but seemed to be confined to ER-positive tumors (9). With regard to the association between the biomarkers of IGF system and the prognosis of breast cancer, studies are limited and have revealed controversial findings suggesting a positive association (10), an inverse association (11), or no clear association of IGF1 or IGFBP3 with adverse outcomes after breast cancer including all-cause or breast cancer-specific mortality and breast cancer recurrence (12, 13). The relatively small sample size of the previous studies may possibly limit the ability to detect modest effects and restricted the generalizability of the results.

In this study, the large prospective sample enabled us to assess the association of IGF1 and prognostic outcomes of breast cancer among specific subgroups by important risk factors and clinical characteristics. We extended the current evidence by providing novel findings that compared with low concentrations of circulating IGF1, high concentrations of IGF1 were independently associated with a decreased risk of all-cause mortality after breast cancer, and such association was further confirmed by a similar association between IGF1 and post-recurrence mortality. Interestingly, the association between IGF1 and all-cause mortality appeared to be consistent across age, BMI, menopausal status, and the most of analyzed clinical characteristics. Surprisingly, we did not observe a statistically significant association of IGF1 with breast cancer-specific mortality or breast cancer recurrence. The lack of a significant association between IGF1 and breast cancer-specific mortality may be mainly due to the relatively low number of breast cancer-specific mortality, which could limit the ability to detect a statistically significant association. In addition, although the lack of a significant association between IGF1 and breast cancer recurrence was not in discordance with previous studies (11, 12), there may be possible confounding factors that influence the observations, which deserve future clinical validations.

Several potential mechanisms may also explain our findings. As a multifunctional peptide, IGF1 has different roles in the initiation and progression of different diseases. In physiological conditions, its antiapoptotic effect can help cell survival; while in pathophysiological conditions, IGF1 can lead to cancer or increment of adipocytes (5, 21–24). It is possible that after the surgery of breast cancer, IGF1 may exhibit a protective function in overall survival *via* its favorable effects on cell survival and metabolic control (5). Additionally, IGF1 plays a protective role on the cardiovascular system through inhibiting the hyperactivity of myocardial Na^+^/H^+^ exchanger-1, protecting the positive inotropic, and exerting antioxidant effects (21, 22). Epidemiological studies also confirmed that low baseline levels of IGF1 increased the risk of fatal ischemic heart disease among elderly men and women independent of prevalent cardiovascular risk factors (23). Moreover, in line with previous findings (23, 24), patients with low concentrations of IGF1 were older and had a higher BMI in this study. Although age and BMI were controlled in the analyses, the underlying pathways may partly account for an increased risk for mortality. Future experimental investigations are needed to elucidate the specific mechanisms underlying our observations.

The strengths of this study include the prospective study design, relatively large sample size, high percentage of complete documents of breast cancer prognosis events, as well as comprehensive measurements of biomarkers of insulin and the IGF axis. However, our study still has several limitations. First, although we have carefully controlled for multiple confounders, residual, and unmeasured confounding and reverse causality may exist. Second, the relatively short follow-up period and low number of events may limit the power of detecting statistically significant associations between the above biomarkers and the prognostic outcomes of interest. For example, given the incidence of breast cancer-specific mortality between the two groups with low or high IGF1 levels, in order to achieve a statistical power of 0.80, the sufficient overall sample size and number of events should be around 2,800 and 80, respectively. Therefore, our findings should be interpreted with caution. Third, in this study, baseline clinical measurements and blood samples were collected from each patient only before the surgical procedure; therefore, no comparison between clinical values before and after the surgery could be obtained, which may miss the subtle dynamic changes of these values during the procedure.

## Conclusions

In this large prospective study, circulating IGF1 was inversely and independently associated with all-cause mortality in invasive breast cancer patients, and this association was consistent among patients with different strata of insulin and clinical characteristics. Our findings suggest the important role of circulating higher levels of IGF1 as a protective predictor of mortality after breast cancer.

## Data Availability Statement

The data is available in coded form at the Comprehensive Breast Health Center, Ruijin Hospital, Shanghai Jiao Tong University School of Medicine. The data that supports the findings of this study is available from the corresponding author on reasonable request: Dr. Kunwei Shen, kwshen@medmail.com.cn.

## Ethics Statement

The studies involving human participants were reviewed and approved by The Medical Ethics Committee of Ruiiin Hospital, Shanghai Jiao Tong University. The patients/participants provided their written informed consent to participate in this study. All participants have given expressed consent for publication of their details. All personal information has been made anonymous.

## Author Contributions

YZ, TW, XC, and KS contributed to the concept and design and contributed to critical revision of the manuscript for important intellectual content. YZ contributed to the drafting of the manuscript. YZ and TW contributed to statistical analysis. XC and KS obtained funding. KS supervised. All authors were involved in administrative, technical, or material support, contributed to the acquisition, and interpretation of data.

## Conflict of Interest

The authors declare that the research was conducted in the absence of any commercial or financial relationships that could be construed as a potential conflict of interest.

## References

[1] Pollak M Insulin and insulin-like growth factor signalling in neoplasia. Nat Rev Cancer. (2008) 8:915–28. 10.1038/nrc2536 19029956

[2] Khandwala HM McCutcheon IE Flyvbjerg A Friend KE. The effects of insulin-like growth factors on tumorigenesis and neoplastic growth. Endocr Rev. (2000) 21:215–44. 10.1210/edrv.21.3.0399 10857553

[3] Jones JI Clemmons DR. Insulin-like growth factors and their binding proteins: biological actions. Endocr Rev. (1995) 16:3–34. 10.1210/edrv-16-1-3 7758431

[4] Grimberg A Cohen P. Role of insulin-like growth factors and their binding proteins in growth control and carcinogenesis. J Cell Physiol. (2000) 183:1–9. 10.1002/(SICI)1097-4652(200004)183:1<1::AID-JCP1>3.0.CO;2-J 10699960 PMC4144680

[5] Puche JE Castilla-Cortázar I. Human conditions of insulin-like growth factor-I (IGF-I) deficiency. J Transl Med. (2012) 10:224. 10.1186/1479-5876-10-224 23148873 PMC3543345

[6] Pollak M. The insulin and insulin-like growth factor receptor family in neoplasia: an update. Nat Rev Cancer. (2012) 12:159–69. 10.1038/nrc3215 22337149

[7] Steuerman R Shevah O Laron Z. Congenital IGF1 deficiency tends to confer protection against post-natal development of malignancies. Eur J Endocrinol. (2011) 164:485–9. 10.1530/EJE-10-0859 21292919

[8] Renehan AG Zwahlen M Minder C O'Dwyer ST Shalet SM Egger M. Insulin-like growth factor (IGF)-I, IGF binding protein-3, and cancer risk: systematic review and meta-regression analysis. Lancet. (2004) 363:1346–53. 10.1016/S0140-6736(04)16044-3 15110491

[9] Endogenous Hormones and Breast Cancer Collaborative Group Key TJ Appleby PN Reeves GK Roddam AW. Insulin-like growth factor 1 (IGF1), IGF binding protein 3 (IGFBP3), and breast cancer risk: pooled individual data analysis of 17 prospective studies. Lancet Oncol. (2010) 11:530–42. 10.1016/S1470-2045(10)70095-4 20472501 PMC3113287

[10] Duggan C Wang CY Neuhouser ML Xiao L Smith AW Reding KW. Associations of insulin-like growth factor and insulin-like growth factor binding protein-3 with mortality in women with breast cancer. Int J Cancer. (2013) 132:1191–200. 10.1002/ijc.27753 22847383 PMC3764990

[11] Kalledsøe L Dragsted LO Hansen L Kyrø C Grønbæk H Tjønneland A. The insulin-like growth factor family and breast cancer prognosis: a prospective cohort study among postmenopausal women in Denmark. Growth Horm IGF Res. (2019) 44:33–42. 10.1016/j.ghir.2018.12.003 30622040

[12] Al-Delaimy WK Flatt SW Natarajan L Laughlin GA Rock CL Gold EB. IGF1 and risk of additional breast cancer in the WHEL study. Endocr Relat Cancer. (2011) 18:235–44. 10.1530/ERC-10-0121 21263044

[13] Hartog H Boezen HM de Jong MM Schaapveld M Wesseling J van der Graaf WT. Prognostic value of insulin-like growth factor 1 and insulin-like growth factor binding protein 3 blood levels in breast cancer. Breast. (2013) 22:1155–60. 10.1016/j.breast.2013.07.038 23968866

[14] Lakhani SR Ellis IO Schnitt SJ Tan PH van de Vijver MJ WHO Classification of Tumours of the Breast, 4th Edition. Lyon: IARC Press (2012).

[15] American Joint Committee on Cancer Updated Breast Cancer Chapter for 8th Edition. (2020). Available online at: https://cancerstaging.org/references-tools/deskreferences/Pages/Breast-Cancer-Staging.aspx (accessed August 5, 2020).

[16] Goldhirsch A Winer EP Coates AS Gelber RD Piccart-Gebhart M Thürlimann B. Personalizing the treatment of women with early breast cancer: highlights of the St Gallen international expert consensus on the primary therapy of early breast cancer 2013. Annu Oncol. (2013) 24:2206–23. 10.1093/annonc/mdt303 23917950 PMC3755334

[17] Hudis CA Barlow WE Costantino JP Gray RJ Pritchard KI Chapman JA. Proposal for standardized definitions for efficacy end points in adjuvant breast cancer trials: the STEEP system. J Clin Oncol. (2007) 25:2127–32. 10.1200/JCO.2006.10.3523 17513820

[18] Christopoulos PF Msaouel P Koutsilieris M. The role of the insulin-like growth factor-1 system in breast cancer. Mol Cancer. (2015) 14:43. 10.1186/s12943-015-0291-7 25743390 PMC4335664

[19] Sachdev D Yee D. The IGF system and breast cancer. Endocr Relat Cancer. (2001) 8:197–209. 10.1677/erc.0.0080197 11566611

[20] Yu H Rohan T. Role of the insulin-like growth factor family in cancer development and progression. J Natl Cancer Inst. (2000) 92:1472–89. 10.1093/jnci/92.18.1472 10995803

[21] Yeves AM Burgos JI Medina AJ Villa-Abrille MC Ennis IL. Cardioprotective role of IGF-1 in the hypertrophied myocardium of the spontaneously hypertensive rats: a key effect on NHE-1 activity. Acta Physiol. (2018) 224:e13092. 10.1111/apha.13092 31595734

[22] Cittadini A Ishiguro Y Strömer H Spindler M Moses AC Clark R Insulin-like growth factor-1 but not growth hormone augments mammalian myocardial contractility by sensitizing the myofilament to Ca2+ through a wortmannin-sensitive pathway. Circ Res. (1998) 83:50–9. 10.1161/01.RES.83.1.50 9670918

[23] Laughlin GA Barrett-Connor E Criqui MH Kritz-Silverstein D The prospective association of serum insulin-like growth factor I (IGF-I) and IGF-binding protein-1 levels with all cause and cardiovascular disease mortality in older adults: the Rancho Bernardo study. J Clin Endocrinol Metab. (2004) 89:114–20. 10.1210/jc.2003-030967 14715837

[24] Juul A Scheike T Davidsen M Gyllenborg J Jørgensen T. Low serum insulin-like growth factor I is associated with increased risk of ischemic heart disease. Circulation. (2002) 106:939–44. 10.1161/01.CIR.0000027563.44593.CC 12186797

