# Supplementary material for: Associations Between Circulating Insulin-Like Growth Factor 1 and Mortality in Women With Invasive Breast Cancer
Source: Front Oncol. 2020 Aug 19;10:1384. doi: 10.3389/fonc.2020.01384 (PMC7466632; doi:10.3389/fonc.2020.01384)

Supplemental material

Table of contents

**Supplemental Table 1.** Medians and ranges of IGF1, IGFBP3, IGF1/IGFBP3 ratio, insulin, and peptide C within low and high levels	………………………………………..2
**Supplemental Table 2.** Incidences of all-cause mortality, breast cancer-specific mortality, and breast cancer recurrence according to IGF1 levels	………………………………………..3
**Supplemental Table 3.** Association of biomarkers of insulin and IGF axis with post-recurrence mortality	………………………………………..4
**Supplementary Figure 1.** Kaplan-Meier curve of post-recurrence mortality according to IGF1 and IGF1/IGFBP3 ratio levels	………………………………………..5

Supplemental Table 1. Medians and ranges of IGF1, IGFBP3, IGF1/IGFBP3 ratio, insulin, and peptide C within low and high levels [a]

	Level	
**Biomarker**	**Low**	**High**
IGF1, ng/mL	120.0 (25.0-157.0)	201.0 (158.0-500.0)
IGFBP3, ug/mL	3.4 (1.0-4.0)	4.5 (4.0-14.6)
IGF1/IGFBP3 ratio, $\times 10^{-3}$	32.1 (11.1-39.4)	48.3 (39.5-102.3)
Insulin, uIU/mL	5.7 (2.4-8.0)	11.2 (8.0-30.5)
C-peptide, ug/L	1.6 (0.9-2.0)	2.5 (2.0-9.8)

[a] There were 2682 participants included in the analysis. The number of missing values was 849 for IGFBP3 and 849 for IGF1/IGFBP3 ratio.

Supplementary Table 2. Incidences of all-cause mortality, breast cancer-specific mortality, and breast cancer recurrence according to IGF1 levels [a]

Outcome	Overall	IGF1		
		Low	High	P value
All-cause mortality	55	37 (2.8)	18 (1.3)	0.007
Breast cancer-specific mortality	43	28 (2.1)	15 (1.1)	0.038
Breast cancer recurrence	157	77 (5.8)	80 (5.9)	0.90

[a] There were 2682 participants included in the analysis.

Supplementary Table 3. Association of biomarkers of insulin and IGF axis with post-recurrence mortality [a]

Category	Breast cancer-specific mortality		
	**Person-years**	**Cases**	**HR (95% CI) [b]**
IGF1			
Low	215	26	1.00
High	261	12	**0.41 (0.20-0.84)**
IGFBP3			
Low	94	7	1.00
High	105	7	1.40 (0.43-4.51)
IGF1/IGFBP3 ratio			
Low	98	11	1.00
High	101	3	**0.21 (0.05-0.84)**
Insulin			
Low	242	17	1.00
High	234	21	1.35 (0.64-2.86)
C-peptide			
Low	250	18	1.00
High	226	20	1.05 (0.51-2.14)

[a] There were 157 participants who had breast cancer recurrence included in the analysis. All mortality were breast cancer-specific mortality. The number of missing values was 73 for IGFBP3 and 73 for IGF1/IGFBP3 ratio.

[b] Data were adjusted for age, BMI, menopausal status (yes or no), tumor size ($\leq$ 2 cm or > 2 cm), lymph node status (positive or negative), chemotherapy (yes or no), radiotherapy (yes or no), endocrine therapy (yes or no), and targeted therapy (yes or no).

Supplementary Figure 1. Kaplan-Meier curve of post-recurrence mortality according to IGF1 and IGF1/IGFBP3 ratio levels

There were 157 participants who had breast cancer recurrence included in the analysis. Log-Rank test: P=0.006 for post-recurrence mortality.

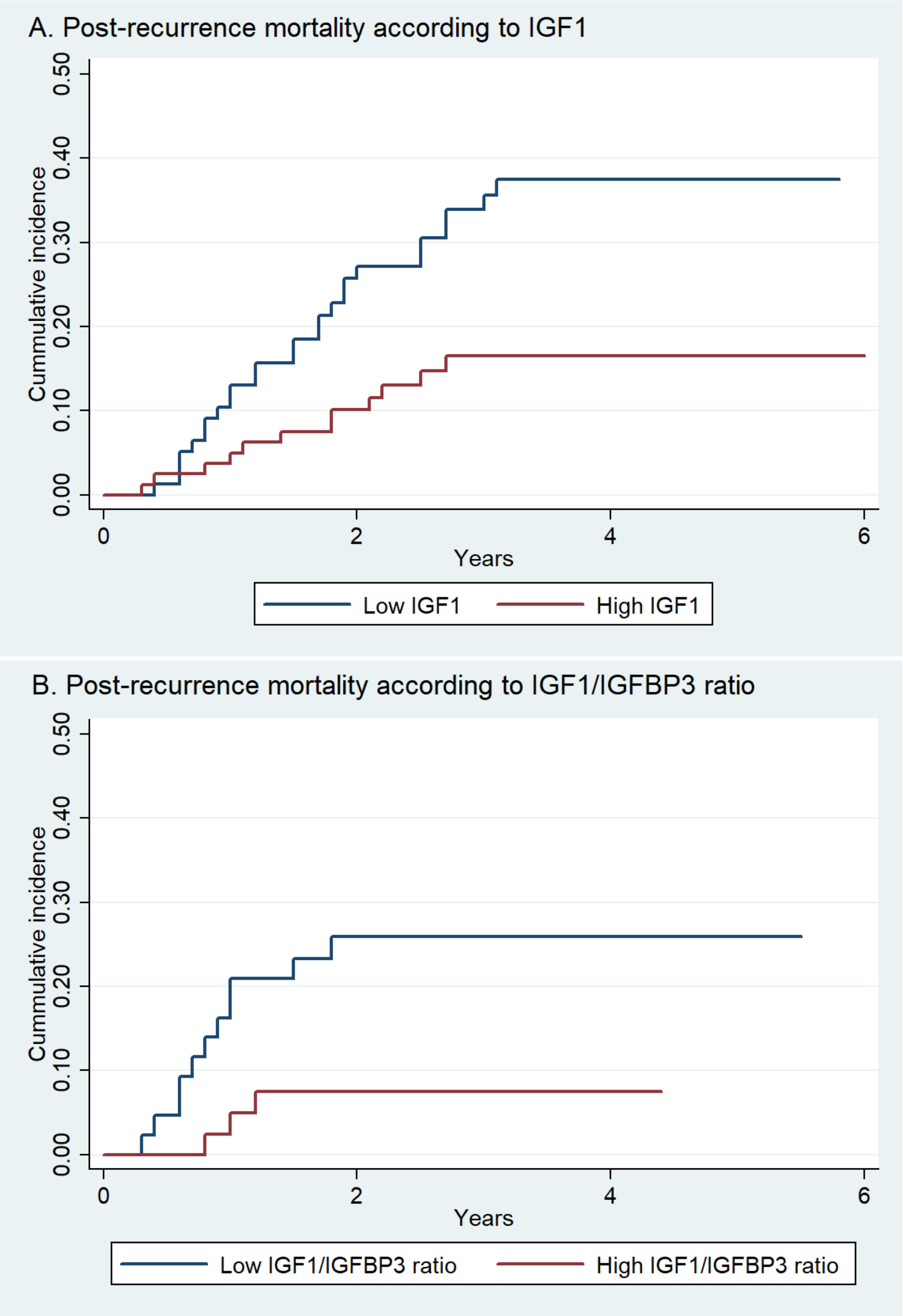

Supplement: Supplementary file 1 [file Data_Sheet_1.PDF]
